# Coculture of tumor organoids with pathogenic microorganisms: a novel system to mimic *in vivo* pathogenic infection

**DOI:** 10.3389/fcimb.2025.1601688

**Published:** 2025-06-30

**Authors:** Xue Zhang, Shulan Sun, Siqi Cheng, Junze Dai, Furong Du, Jingrui Wang, Dan Wei, Yichao Yan, Yefu Liu

**Affiliations:** ^1^ Central Laboratory, Cancer Hospital of Dalian University of Technology, Liaoning Cancer Hospital and Institute, Shenyang, Liaoning, China; ^2^ China Medical University, Shenyang, Shenyang, Liaoning, China; ^3^ Graduate School, Dalian Medical University, Dalian, Liaoning, China; ^4^ Department of Medicine, Kingbio Medical Co., Ltd., Chongqing, China; ^5^ Department of Pharmacy, Liaoning Vocational College of Medicine, Shenyang, Liaoning, China; ^6^ Department of Gastroenterological Surgery, Peking University International Hospital, Beijing, China; ^7^ Department of Hepatobiliary and Pancreatic Surgery, Cancer Hospital of Dalian University of Technology, Liaoning Cancer Hospital and Institute, Shenyang, Liaoning, China

**Keywords:** tumor organoids, microorganisms, pathogenic infection, coculture, interactions

## Abstract

Since the early 20^th^ century, there has been extensive discussion on the intricate relationship between pathogenic infection and tumors. However, most studies on host-pathogen interactions are performed based on the *in-vitro* culture, immortalized cell lines or animal experiments. A significant challenge lies in accurately establishing a coculture model between tumors and pathogens under the three-dimensional (3D) context. Recently, the hybrid model system that incorporates 3D tumor organoids and two-dimensional cell lines have been gradually used to analyze the intricate relationship between pathogens and tumors, and several coculture techniques for tumor organoids and pathogens have also been developed. Therefore, this study systematically reviewed the preparation and identification of tumor organoids, coculture techniques with pathogens, and their clinical applications, aiming to further understand and simulate the interaction mechanism between the hosts and pathogens.

## 1 Introduction

Organoids serve as a transitional model between *in vitro* cancer cell lines and xenografts, offering a unique approach to study cancer biology. Differing from traditional cell culture, the organoid model can preserve cell-cell and cell-matrix interactions by cultivating cancer cells under the three-dimensional (3D) context (Veninga and Voest, 2021; Xu et al., 2022), more closely resembling the characteristics of the original tumor (McCauley and Wells, 2017; Ouchi et al., 2019; Kim et al., 2020). Organoids are classified based on the cellular source, including pluripotent stem cells (PSCs), adult stem cells (ASCs), and patient-derived tumor organoids (PDTOs) (Driehuis et al., 2020; Tindle et al., 2021; Li et al., 2023) (**Table 1**), among which the PDTOs are small tissue spheroids and are generated following tumor resection (Walsh et al., 2017; Rosenbluth et al., 2020). The advent of PDTOs has enabled the implementation of patient-specific drug screening, personalized treatment, and identification of prognostic biomarkers and mechanisms of drug resistance (Neal and Kuo, 2016).

**Table 1:** Advantages and limitations of the organoid culture methods.

Coculture methods	Stem cell-derived organoids	Patient-derived tumor organoids
Wild-type cell culture	+	+
Preinvasive cancer models	+	+
Invasive cancer models	+	+
Metastatic cancer models	+	+
Cost	$$	$$
Time	+++	++
Success rates	Low	Medium
Throughput therapies	High	Medium

+, denotes 1 month or less; ++, 1–2 month; +++, often more than several months.

The intricate relationship between infectious diseases and cancers has been extensively studied since the early 20^th^ century. In 2012, approximately 2.2 million new cancer cases were attributed to infections, among which helicobacter pylori (*H. pylori*), human papillomavirus (HPV), hepatitis B virus (HBV), hepatitis C virus (HCV), and Epstein-Barr virus (EBV) play important roles (Qu et al., 2021). Previous studies have confirmed that tumorigenesis is closely associated with a variety of pathogenic microorganisms comprising a heterogeneous assemblage of bacteria, fungi, protozoa, viruses, and phages (Maiuri et al., 2017; Purcell et al., 2017; Tsoi et al., 2017; Kidane, 2018; Tsay et al., 2018; Zhang et al., 2018; Allen and Sears, 2019; Allen et al., 2019; Brennan and Garrett, 2019; Parhi et al., 2020; Valguarnera and Wardenburg, 2020; DeStefano Shields et al., 2021; Tsay et al., 2021) (**Table 2**). Nejman et al. undertook an exhaustive examination of the microbiomes in 1,526 tumors (breast, lung, ovarian, pancreatic, melanoma, bone, and brain tumors) and their corresponding normal tissues across 7 distinct cancer types. The findings revealed that each tumor category exhibited a distinct microbiome profile, with breast cancer demonstrating a notably abundant and varied microbiome (Plummer et al., 2016; Nejman et al., 2020). This indicates that coculturing pathogenic microorganisms with tumor organoids offers a new approach for diagnosis, prognostic prediction, and treatment decision in cancer. Although bacterial therapy has shown a greater promise in cancer treatment over the last decade due to its ability to lyse the tumor cells and deliver therapeutic products, the potential cytotoxicity of bacteria for healthy tissues and their inability to entirely lyse cancerous cells poses challenges for cancer treatment (Sepich-Poore et al., 2021; Soleimani and Javadi, 2022). Hence, the investigation into pathogenic microorganisms is crucial for understanding the mechanisms of tumorigenesis and promoting the development of innovative vaccine technologies.

**Table 2:** Common mechanisms of pathogen-induced tumorigenesis across tumor types.

Tumor types	Pathogen types	Mechanisms
Colorectal cancer	*Bacteroides fragilis*	NF-κB-STAT3, IL-17 production (Valguarnera and Wardenburg, 2020);
		Methylation (Maiuri et al., 2017; DeStefano Shields et al., 2021);
		Activate CEC Wnt signaling, induce c-Myc expression, and amplify CEC proliferation (Valguarnera and Wardenburg, 2020);
		Upregulate CEACAM, and downregulate MUC2 (Allen et al., 2019)
	*Escherichia coli*	DNA inter-strand crosslinks, DNA double-strand breaks, chromosomal aberrances, and cell cycle arrest (Allen and Sears, 2019)
	*Fusobacterium nucleatum*	TLR4-NFκB, Wnt/β-catenin (Brennan and Garrett, 2019)
	*Streptococcus gallolyticus*	High NF-κB and IL-8 messenger RNA tissue expression (Zhang et al., 2018)
	*Peptostreptococcus stomatis*	Acidity and hypoxia (Purcell et al., 2017)
	*Peptostreptococcus anaerobius*	ROS accumulation promoting bacterial colonization and cellular proliferation respectively (Tsoi et al., 2017)
Gastric cancer	*Helicobacter pylori*	Produce ROS and nitrogen species, trigger single-strand DNA breaks and/or induce the NF-κB pro-inflammatory pathway that can trigger double-strand DNA breaks (Kidane, 2018)
*Lung cancer*	*Veillonella parvula*	Upregulate IL-17, PI3K-AKT, MAPK and ERK pathways as well as IL-6/IL-8 (Tsay et al., 2021)
	*Streptococcus*	Upregulate the ERK and PI3K pathways (Tsay et al., 2018)
Breast cancer	*Fusobacterium nucleatum*	Enhanced tumor growth inhibited by antibiotics (Parhi et al., 2020)

Certain bacteria can induce cancers in diverse organs and tissues, including lung, liver, colorectum, kidney, cervix, brain, gastrointestinal tract, etc (Ward et al., 1994; Fukuda et al., 2002; Kobayashi et al., 2005; Boleij et al., 2011; Zhan et al., 2011; Akkari et al., 2012; Kostic et al., 2012; Arzumanyan et al., 2013; Boleij and Tjalsma, 2013; Buc et al., 2013; Bonnet et al., 2014; Hua-Feng et al., 2015; Oh et al., 2015; Amieva and Peek, 2016; Zhu et al., 2016; Bullman et al., 2017; Di Domenico et al., 2017; Drewes et al., 2017; Chouhan et al., 2019; Wang et al., 2020; Zhang et al., 2020; Kostyusheva et al., 2021; Arcia Franchini et al., 2022; Bessède and Mégraud, 2022; Campbell, 2022; Wasunan et al., 2022; Guo et al., 2023; Seelbinder et al., 2023; Abbas et al., 2024; Zhang et al., 2024; Incognito et al., 2025; Peng et al., 2025) (**Table 3**). These bacteria contribute to tumorigenesis or malignant progression through various mechanisms (Wong and Yu, 2023; Kwon et al., 2024). Here, we systematically reviewed the preparation and identification of tumor organoids, coculture techniques of tumor organoids and pathogenic microorganisms, and their clinical application.

**Table 3:** Common pathogenic microorganisms with cancer-related risk.

Cancer types	Pathogenic microorganisms
Gastric cancer	*H. pylori* (Amieva and Peek, 2016), *Epstein-Barr virus* (Bessède and Mégraud, 2022), *Mycobacterium* (Chouhan et al., 2019), *Eggerthia catenaformis* (Wang et al., 2020)
Colorectal cancer	*pks+ Escherichia coli* (Buc et al., 2013; Bonnet et al., 2014), *Fusobacterium nucleatum* (Kostic et al., 2012; Bullman et al., 2017; Drewes et al., 2017), *Streptococcus gallolyticus* (Boleij et al., 2011; Boleij and Tjalsma, 2013)
Cervical cancer	HPV (Guo et al., 2023; Abbas et al., 2024), *Prevotella* (Zhang et al., 2024; Peng et al., 2025), *Lactobacillus crispatus* (Oh et al., 2015; Incognito et al., 2025), *Chlamydia trachomatis* (Zhu et al., 2016; Arcia Franchini et al., 2022)
Nasopharyngeal Carcinoma	*Epstein-Barr virus* (Zhang et al., 2020)
Hepatocellular Carcinoma	HBV (Kostyusheva et al., 2021; Campbell, 2022), HCV (Akkari et al., 2012; Arzumanyan et al., 2013), *Bacillus subtilis* (botany) (Wasunan et al., 2022), *Escherichia* sp*iralis* (genus of bacteria) (Ward et al., 1994)
Gallbladder cancer	*Salmonella* (Di Domenico et al., 2017), *H. pylori* (Fukuda et al., 2002; Kobayashi et al., 2005)
Lung cancer	*Chlamydia pneumoniae* (Zhan et al., 2011; Hua-Feng et al., 2015), *Candida* (Seelbinder et al., 2023)

## 2 Preparation and characterization of tumor organoids

**Figure 1:**
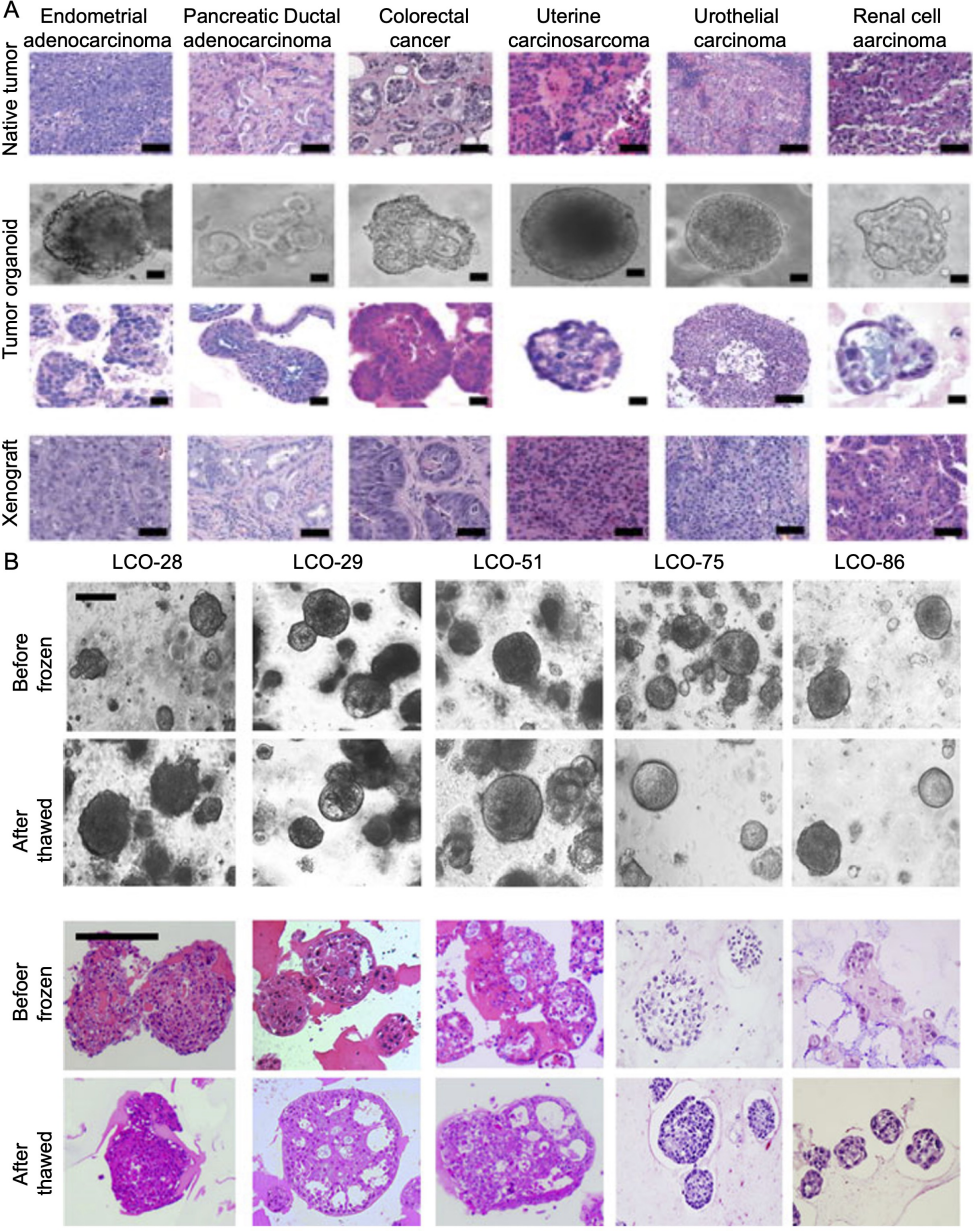
**(A)** Natural tumor specimens and their derived tumor organoids and xenografts (detailed procedure can be found in reference (Pauli et al., 2017). **(B)** Bright field microscopy images and H&E-stained images of LCOs before freezing and after thawing. After the thawing test on cryopreserved organoids, the morphology of organoids and the histologic features of original tissues were reconstituted. Scale bar, 200 μm. Information about the LCOs in these images: LCO-28, squamous cell carcinoma; LCO-29, large cell carcinoma; LCO-51, adenocarcinoma; LCO-75, small cell carcinoma; LCO-86, adenosquamous carcinoma (detailed procedure can be found in reference (Kim et al., 2019). LCO, Lung cancer organoids.

In recent years, PDTOs have been widely used to study various cancer types, including pancreatic cancer (Boj et al., 2015), prostate cancer (Gao et al., 2014), liver cancer (Broutier et al., 2017), bladder cancer (Lee et al., 2018), breast cancer (Sachs et al., 2018), ovarian cancer (Kopper et al., 2019) and gastric cancer (Yan et al., 2018). Cancer is an extremely complex disease, and its heterogeneity is manifested by the fact that the same cancer subtype may vary significantly among the patients, such as the cell shape, size, and gene expression (**Figure 1**). The quality control of different tumor organoids, especially the stable expression of markers, plays a very important role in identifying successful establishment. The morphology and culture conditions of tumor organoids have been reported in several studies (Karthaus et al., 2014; Yoshida, 2020; Jeong et al., 2023). To provide a basis for standardized quality control of tumor organoids, we summarized the markers applied in the identification of tumor organoids (Karthaus et al., 2014; Boj et al., 2015; van de Wetering et al., 2015; Broutier et al., 2017; Lee et al., 2018; Sachs et al., 2018; Yan et al., 2018; Kopper et al., 2019; Jacob et al., 2020b; Lõhmussaar et al., 2021; Tao et al., 2022; Ou et al., 2023; Wang et al., 2023; He et al., 2025) (**Table 4**).

**Table 4:** Identification of tumor organoid markers.

Cancer types	Tissue source	Markers
Prostate cancer (Karthaus et al., 2014)	Prostate luminal cells	Basal prostate markers: p63 and CK5; Basal (outer) layer: CK8
Ovarian cancer (Kopper et al., 2019; Tao et al., 2022)	Surgical tissue and/or drainage of ascites/pleural effusion	Epithelial markers: CK8, CK18, E-cadherin; High-grade serous ovarian cancer markers: PAX8, p53, CK7
Pancreatic cancer (Boj et al., 2015; He et al., 2025)	Surgical or biopsy tissue	Duct cell markers: Ki-67, CD68, and CK19
Moderate/highly differentiated hepatocellular carcinoma (HCC) (Broutier et al., 2017)	Liver tissue (of donor origin) from patients undergoing surgery	AFP and GPC3
Cholangiocarcinoma (CC) (Broutier et al., 2017)	Liver tissue (of donor origin) obtained from patients undergoing surgery	EPCAM, KRT19 or S100A11
Combined HCC/CC (Broutier et al., 2017)	Liver tissue (of donor origin) from patients undergoing surgery	Markers that express both HCC and CC
Bladder cancer (Lee et al., 2018)	Surgical tissue	Urinary tract epithelial cell markers: CK7; basal epithelial markers: CK5; Luminal epithelial markers: CK8
Breast cancer (Sachs et al., 2018)	Surgical tissue	ERα, PR, and HER2
Gastric cancer (Yan et al., 2018)	Surgical tissue	Gastric markers: MUC5AC, PGC, SST, MUC6, TFF1, TFF2
Cervical cancer (Lõhmussaar et al., 2021)	Healthy endocervical and extracervical tissues dissected from the cervical canal in women undergoing total hysterectomy	Endocervical tissues and organoids: secretory cell transcriptional marker PAX8; Ectocervical organoids: KRT14-positive basal-like cells and differentiated KRT13-positive layers; Markers to confirm the origin of endocervical lining and to determine the extent of disease: PAX8 and MKI67
Lung adenocarcinoma (Wang et al., 2023)	Biopsy or surgical excision of primary or metastatic lesions to obtain fresh tissue and collection of malignant fluid samples using sterile drainage bags	CK7, TTF-1 and Napsin A
Squamous cell carcinoma (Wang et al., 2023)	Biopsy or surgical excision of primary or metastatic lesions to obtain fresh tissue and collection of malignant fluid samples using sterile drainage bags	P40, P63 and CK5/6
Small cell lung cancer (Wang et al., 2023)	Biopsy or surgical excision of primary or metastatic lesions to obtain fresh tissue and collection of malignant fluid samples using sterile drainage bags	Neuroendocrine markers: CD56, synaptophysin, CgA and TTF-1
Colorectal cancer (van de Wetering et al., 2015)	Surgical tissue	KI67, OLFM4, KRT20 and Alcian blue
Melanoma (Ou et al., 2023)	Obtained from patients receiving treatment	HMB-45, α-SMA, vimentin and ICAM-1
Glioblastoma (Jacob et al., 2020b)	Surgically resected fresh glioblastoma tissue	Glial cell markers: GFAP and S100B; Mature neuron marker DCX and neural progenitor and glioma stem cell markers NESTIN, BLBP, HOPX, SOX2 and OLIG2

## 3 Development of organoid coculture techniques with pathogens

Coculture techniques play pivotal roles in the examination of host-pathogen interactions and the simplification of *in vivo* systems. The predictive capacity of cell culture-based assays is constrained by their inability to replicate the intricate organ complexity and inter-tissue communication present *in vivo* (LeSavage et al., 2022). The advent of microphysiological systems, exemplified by organoid cocultures, has achieved great progress in the fields of stem cell biology, disease modeling, and host-pathogen interactions. Nevertheless, there still exist intricate microbe-disease relationships. Hence, it is very necessary to develop simplified and meaningful approaches to model host-microbe interactions, and to visualize and analyze the mechanisms of bacterial adhesion and internalization at the microscopic level.

**Figure 2:**
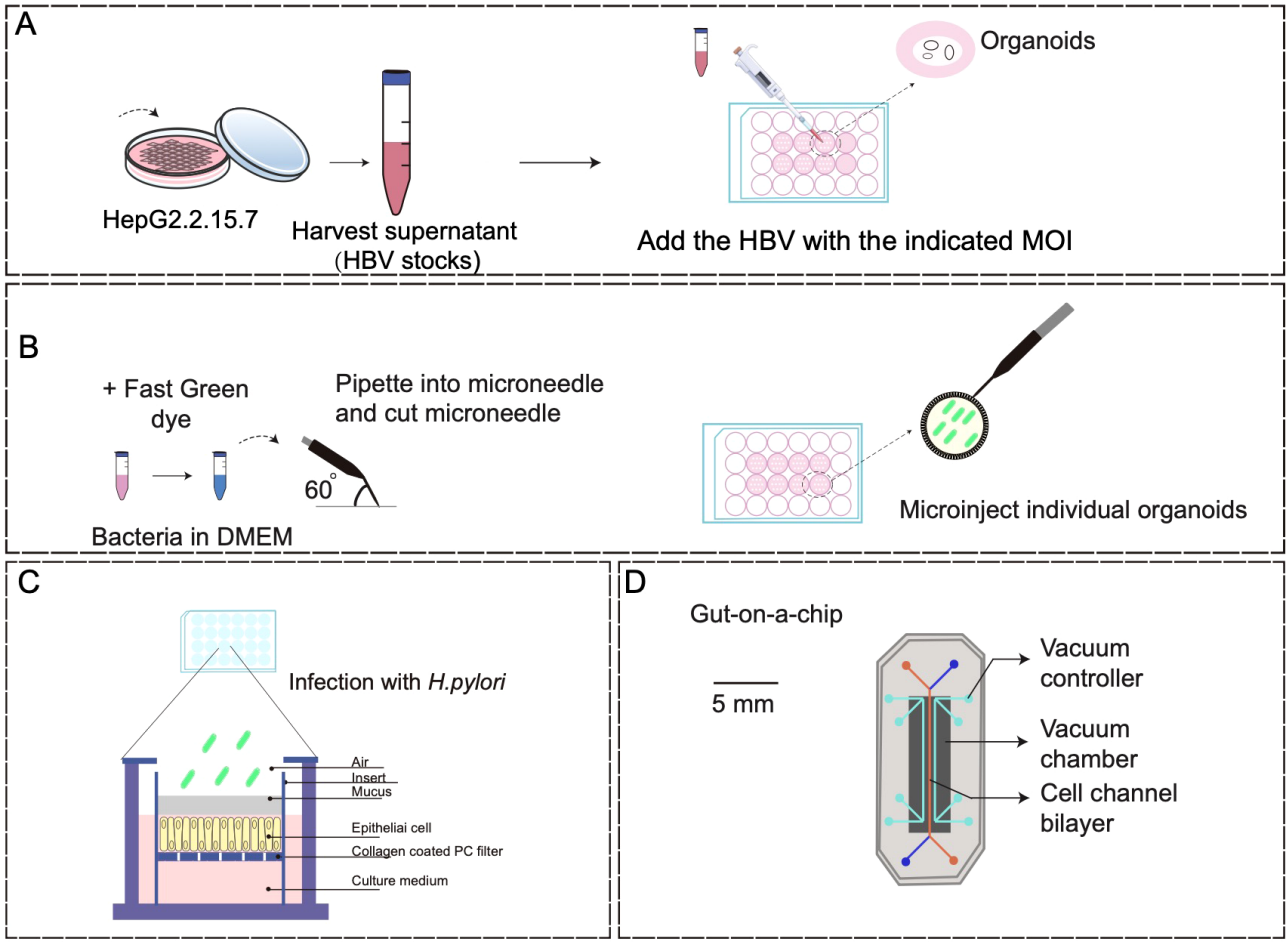
Coculture models of organoids with pathogenic microorganisms. **(A)** A schematic diagram of direct infection of HepG2.2.15.7 cells with HBV stock solution (detailed procedure can be found in reference (Nie et al., 2018). **(B)** Organoid microinjections (detailed procedure can be found in reference (Puschhof et al., 2021). **(C)** Human gastric mucosal columnar epithelium was regenerated using the air-liquid interface culture method (detailed procedure can be found in reference (Boccellato et al., 2019). **(D)** Human gut-on-a-chip inhabited by microbial flora (detailed procedure can be found in reference (Kim et al., 2012).

There are several methods for cocultures, such as direct coculture of viruses with organoids and injection of microorganisms into the organoid lumen. In the study of Nie et al., the HBV-containing supernatant of HepG2.2.15.7 cells, a HepG2.2.15 clone producing a higher level of HBV, was utilized to coculture with human induced pluripotent stem cell (hiPSC)-liver organoids, hiPSCs-hepatic-like cells, HepG2-tet-Na+-taurocholate cotransporting polypeptide organoids, and primary human hepatocytes in 24-well plates at a specific ratio (**Figure 2A**) (Nie et al., 2018). The harvested cells were then subjected to HBV covalent closed circular DNA (cccDNA) assay after infection for 10–20 days. This study successfully developed a stable HBV infection model through direct coculture of pathogens and PSCs-induced organoids. However, the coculture period is long, and organoids for passage and clonal growth following exposure to pathogens were limited after long-term culture.

With the development of modern biotechnology, the microinjection of microorganisms into the organoid lumen has further enhanced the efficacy of coculture techniques (**Figure 2B**). The utility of microinjection lies in its capacity to accurately regulate the specific physiological localization of bacteria, although it is not conducive to conducting extensive infection studies. Furthermore, the adoption of transwell-based cell culture methods for investigating bacterial interactions with physiological tissue barriers is steadily increasing (**Figure 2C**). This method offers the benefit of ensuring consistent exposure of individual cells to microorganisms, but the absence of spatial and environmental protection in bacterial compartments results in reduced viability of specialized anaerobes or unregulated proliferation of other bacterial strains (Boccellato et al., 2019).

Microfluidic organoids-on-a-chip, derived from host tissue cells, offers a valuable tool for *in vitro* organ mimicry. This allows researchers to manipulate various cellular, molecular, chemical, and biophysical parameters in a controlled manner, either individually or in combination, to study their impact on the development and progression of human cancers, as well as the efficacy of therapeutic interventions (Kim et al., 2012; Bhatia and Ingber, 2014; Benam et al., 2016; Kasendra et al., 2018) (**Figure 2D**). Organoids-on-a-chip is a microfluidic cell culture device made of materials, such as optically transparent plastics, glass, or flexible polymers like polydimethylsiloxane, which contains perfused hollow microchannels filled with living cells (Kim et al., 2012; Kasendra et al., 2018). For instance, humans exhibit a significant vulnerability to enterohemorrhagic *Escherichia coli* (EHEC) infection, whereas mice display a relatively low susceptibility to this pathogen (Tovaglieri et al., 2019). Through the utilization of human colon microarray microfluidic culture technology, researchers simulated EHEC infection-induced epithelial damage in the human colon and found that exposure to metabolites originating from the human intestinal microbiomes resulted in more pronounced epithelial damage compared to mice (Tovaglieri et al., 2019). This study employed a multi-omics approach to identify 4 human microbiome metabolites as the mediators of this effect, including 4-methylbenzoic acid, 3,4-dimethylbenzoic acid, hexanoic acid, and heptanoic acid. Previous research on human host-microbiome-pathogen interactions primarily relied on the relevant genomic or macrogenomic studies, posing great challenges to establish causality in human pathogenesis (Surana and Kasper, 2017). The *in vitro* system described in this study demonstrates species-specificity and highlights the advantages of coculture systems based on the organoids-on-a-chip compared with organoid cultures alone. Additionally, Sun et al. presented an oncolytic virus (OV) evaluation system using microfluidic organ-on-a-chip systems and patient-derived hypopharyngeal and breast cancer organoids, and found that AD4-GHPE, a novel OV, had three antitumor mechanisms: tumor-specific cytotoxicity, a reduction in PD-L1 expression in tumor cells to increase CD8+ T-cell activity, and granulocyte-macrophage colony-stimulating factor secretion (Sun et al., 2025). This evaluation system based on tumor organoids is efficient and reliable, offering a personalized OV treatment recommendation for patients and providing industrialized and standardized research ideas for OV development.

## 4 Application of tumor organoids cocultured with pathogenic microorganisms

Coculture techniques have been widely utilized in the field of biology to investigate interactions between various cell populations, or cells and pathogenic microorganisms (Goers et al., 2014). Under this context, we focus on the cocultivation of pathogenic microorganisms and tumor cells. Traditional coculture systems using the cancer cell lines, such as direct coculture, indirect coculture, and co-immobilized mixed culture, are complex and lack versatility, and are unable to accurately replicate the host environment. In contrast, tumor organoid coculture models offer a more effective means of simulating the intricate interactions that occur within tumor tissues.

### 4.1 Brain tumor organoids and viral infections

Gliomas are the most common and lethal primary malignant adult brain tumors, in which glioblastomas are the most common (Zavala-Vega et al., 2019). EBV, a member of the herpesviridae family, was the first oncolytic virus to be described. Since then, several viruses associated with cancer have been identified (Cobbs, 2013; Lisyany et al., 2019).

In 2013, Lancaster and Knoblich developed a methodology for culturing brain organoids comprising multiple brain regions. As the organoid develops, cerebrospinal fluid similar to that in the lateral ventricles is found within the neuroepithelial buds. Concurrently, the neuroepithelial cells undergo additional differentiation and migration towards the outer layers, culminating in the formation of brain organoid cultures with various brain regions, including the forebrain, choroid plexus, hippocampal region, and prefrontal lobe (Lancaster et al., 2013). In 2014, Lancaster et al. developed a human PSC-derived 3D organoid culture system, known as brain organoids, which can generate various distinct and interconnected brain regions (Lancaster et al., 2013). Importantly, these brain organoids have been effectively utilized in modeling the pathogenesis of primary microcephaly through lentiviral shRNA targeting of CDK5/RAP2-dependent pathways.

The human brain is frequently susceptible to viral infections, and numerous viral families contain neurotropic viruses (Ruiz-Guillen et al., 2017; Tavcar et al., 2021). Neurological infections can cause central nervous system disorders, consequently leading to fatality or long-term consequences (Hopkins et al., 2021). Human cytomegalovirus (HCMV) infection is linked to human glioblastoma, but the precise mechanisms of infection remain incompletely elucidated. Dong et al. utilized the tissues from the glioblastoma margin to establish glioblastoma organoids (GBOs), and then cocultured the GBOs with HCMV after treatment with a 2,5-dimethylpyrrolizidine benzoic acid derivative, an EphA2 antagonist (Dong et al., 2023). The results revealed that EphA2 might serve as a potential therapeutic target for inhibiting HCMV infection in glioblastoma cells. The use of brain organoids offers a versatile human cellular platform for investigating cellular susceptibility, disease mechanisms, and therapeutic interventions (Jacob et al., 2020a). With the development of organoids cocultured with pathogenic microorganisms, the potential mechanism of brain tumors may be further illuminated.

### 4.2 Lung organoids and viruses

Lung cancer stands as the leading cause of cancer-related death worldwide (Hirsch et al., 2017). Although organoids established from human lung cancer resections and metastatic biopsies can preserve tumor histopathological and molecular features (Kim et al., 2019), there are rare studies regarding the association between lung cancer and infection based on the organoid platform. In view of this, we mainly investigated the relationship between organoids and respiratory viruses.

Recently, lung organoids have shown their suitability as the models for studying respiratory viruses. In a previous study, respiratory syncytial virus (RSV) and human parainfluenza virus (HPIV) were found to successfully infect human airway organoids (Porotto et al., 2019), which might serve as a versatile model for studying hereditary, malignant, and infectious pulmonary diseases (Sachs et al., 2019). There are also studies that use differentiated airway organoids to predict the infectivity of emerging respiratory viruses, including human and avian influenza viruses and zoonotic coronaviruses (Hui et al., 2018; Zhou et al., 2018; Han et al., 2021; Lamers et al., 2021). Importantly, the lung organoid platform can be used to screen therapeutic drugs and anti-microbial drugs (Sachs et al., 2019).

Regarding respiratory infectious diseases, virologists are trying to use organoid models as platforms to understand the mechanisms of viral infection, cell deregulation and drug screening, but there is still much to do in bacterial and parasitic infections (Fonseca et al., 2017). Heo et al. utilized organoids to illustrate the interaction of a human protozoan parasite, Cryptosporidium, with intestinal and lung epithelia that were considered as the two major sites of infection (Heo et al., 2018). After injection of Cryptosporidium oocysts into the organoid lumen, the parasite propagated within the organoids and completed its life cycle. Additionally, this study also highlighted the importance of interferon-I signaling in response to Cryptosporidium infection through transcriptomic analysis (Heo et al., 2018). In the future, we believe that cocultures of pathogens with lung organoids will be better established to understand and predict human infectious diseases.

### 4.3 Nasopharyngeal carcinoma organoids and EBV

Nasopharyngeal carcinoma (NPC) is a highly aggressive malignant tumor. Its etiology is multifactorial, in which EBV infection may be a major pathogenic factor (Chen et al., 2019). In 2022, Wang et al. successfully cultured NPCOs from a total of 77 samples, including 34 primary samples, 28 recurrent samples, and 15 samples of normal mucosa. The corresponding success rates of NPCOs were 47.06%, 81.25%, and 86.5%, respectively (Wang et al., 2022). All non-keratinizing NPCO samples exhibited positive for EBV-encoded small RNA (EBER) and negative for CK7. The recurrent NPCOs demonstrated increased expression of stem cell markers, including BMI-1, CD44, and CD133. Furthermore, the recurrent NPCOs could be successfully cultured up to the 4^th^ generation and underwent multiple freeze-thaw cycles, unlike primary NPCOs which proved challenging to culture. Through histological staining, immunohistochemistry, and EBER *in situ* hybridization (ISH) assays, it was observed that NPCOs could retain the pathological characteristics of the original tumors and EBV infection status to a significant extent.

### 4.4 Gastric cancer organoids and *H. pylori*

*H. pylori* is an organism related to ulcer disease and gastric cancer, and its oncogenic actions fully reflect the intricate interplay between human cells, microorganisms, and the environment (Wroblewski et al., 2010). *H. pylori* infection can cause chronic inflammation of the gastric mucosa, resulting in gastric mucosal cell changes and atrophy to promote development of precancerous lesions and cancer.

Over a decade ago, human gastric organoids (hGOs) were successfully established utilizing gastric cancer tissue, cancerous site tissue, and induced PSCs (Kalabis et al., 2012; McCracken et al., 2014; Broda et al., 2019; Holokai et al., 2019). In 2014, McCracken et al. successfully developed a 3D hGO *in vitro* through directed differentiation of human PSCs (McCracken et al., 2014). The formation of these organoids depends on the regulation of various signaling pathways, including FGF, Wnt, BMP, retinoic acid, and EGF. The development of hGOs follows similar molecular and morphogenetic stages as observed in the mouse gastric development. In 2019, Holokai et al. demonstrated that *H. pylori* can induce the expression of the immune checkpoint molecule PD-L1 (CD274) via the Shh signaling pathway in a human organ culture model (Holokai et al., 2019). This study employed a coculture system involving patient-derived organoids infected with *H. pylori* and autologous immune cells to develop the therapy of *H. pylori* and PD-1 inhibitors and explore the protective role of PD-L1 against bacterial infection. In 2023, Wuputra et al. developed an organoid model of *H. pylori* infection by constructing a cytotoxin-associated gene A-GFP-tagged strain of *H. pylori* and infecting gastric organoids through microinjection (Wuputra et al., 2023). This resulted in the successful creation of a gastric organoid model capable of simulating *H. pylori* infection *in vivo*. To elucidate the functions of HDGF and TNFα secreted by *H. pylori*-infected tumor organoids, this study prepared recombinant HDGF and TNFα, and assessed the cytotoxicity and invasiveness of gastric cancer organoids. The findings suggest that HDGF and TNFα act as independent signaling molecules in the progression of gastric cancer infected by *H. pylori*.

The timeline from *H. pylori* infection to gastric atrophy, intestinal metaplasia and intraepithelial neoplasia may be months to years long (Piazuelo et al., 2021). During this period, the loss of acid-secreting parietal cells makes the stomach in a relatively hypochlorous environment, promoting changes in the composition of the gastric microbiota (Li and Perez Perez, 2018; Lahner et al., 2020; Barra et al., 2021). In humans with chronic gastritis, *Prevotella*, *Streptococcus*, *Pseudomonas*, Sphingobacterium, *Bacillus*, and *Fusobacterium* have also been found in normal mucosa adjacent to tumors. However, *H. pylori* remains an organism consistently identified at different stages of progression (Barra et al., 2021). The utilization of organoid models that are more sophisticated than the conventional models, such as cell lines, would enhance research on gastric epithelial repair, the function of gastric hormones, and the mechanisms of vaccine-induced protection.

### 4.5 Hepatocellular cancer organoids and HBV

HBV infection is the primary etiological factor for chronic cirrhosis and HCC (Di Bisceglie, 2009; MacLachlan and Cowie, 2015; An et al., 2018). The infection and replication of HBV are characterized by high specificity in host species and organs, which is believed to govern the intricate interplay between the immune response and virus-specific factors to culminate in the development of HCC. Epidemiological investigations have predominantly elucidated the molecular pathways involved in HBV-induced HCC, and genome-wide analyses of viral and host features are conducted (Fattovich et al., 2008; El-Serag, 2012; Fujimoto et al., 2012; Huang et al., 2012; Jiang et al., 2012; Ji et al., 2014; Shibata and Aburatani, 2014; Nantasanti et al., 2016; Cancer Genome Atlas Research Network, 2017; Sartorius et al., 2019; Sagnelli et al., 2020). Moreover, HCC cell lines are utilized *in vitro* studies (Zhang et al., 2014; Thomas and Liang, 2016). Nevertheless, the lack of appropriate animal or *in vitro* model systems for studying HBV infection poses a significant challenge due to the virus-specific host and cell type preferences. Chimpanzees are currently the sole animal model capable of supporting the entire HBV replication process, as they exhibit distinctly different gene expression profiles compared with primary cells (Protzer, 2017).

In 2021, a research team successfully cultured a liver organoid-derived primary *in vitro* HBV infection model from a healthy donor (De Crignis et al., 2021). These organoids were demonstrated to generate HBV cccDNA and HBeAg, and express intracellular HBV RNA and proteins, consequently producing infectious HBV. HBV-infected hepatocyte organoid platforms hold promise for drug screening to assess anti-HBV efficacy and drug-induced toxicity. Additionally, this study also utilized lentivirus to create transgenic organoid lines with integrated copies of HBV, contributing to viral production and HBV transcriptional research. Due to the diverse nature and immunosuppressive conditions, a significant majority (80-90%) of HCC patients do not exhibit objective responses to immunotherapy. Zou et al. developed chimeric antigen receptor T cells targeting HBV surface proteins (HBV-car-T cells) and personalized tumor-reactive CD8^+^ T cells (Zou et al., 2021). Subsequently, a coculture system involving autologous HBV^+^ HCC organoids and T cells was employed to assess their anti-tumor efficacy and mechanisms. Based on the microfluidic chip, a liver organoid system containing CD8^+^ T cells and ASCs was developed (Natarajan et al., 2022). This microfluidic coculture system supported the capability of targeted killing liver organoids with HCV non-structural protein 3-specific peptides under the circumstance of patient-derived KLVALGINAV CD8^+^ T cells. Furthermore, this study further underscored the innovative utility of the co-culture system for investigating the molecular mechanisms underlying the adaptive immune response to HCV in an *in vitro* model employing primary human cells.

### 4.6 Cervical cancer organoids and HPV

Over 90% of cervical cancer patients are attributed to high-risk HPV infection, particularly HPV-16 and HPV-18. High-risk HPV is known to cause cervical cancer through the expression of its E6/E7 proto-oncoproteins (Pal and Kundu, 2020). The squamocolumnar junction (SCJ) is the primary site of HPV infection (Rajendra and Sharma, 2019). Nevertheless, the absence of human-derived *in vitro* models for the SCJ has hindered the research on precancerous lesions and HPV-related cancers.

In 2020, researchers successfully generated organoids derived from the normal SCJ region using stromal gel 3D culture technology. These SCJ organoids primarily consisted of squamous cells in a compact structure, with some mucin-secreting uterine cervical canal cells present alongside the squamous cell population. Transcriptome analysis revealed elevated expression levels of SCJ marker genes in these organoids compared to immortalized cervical cell lines originating from non-SCJ regions (Maru et al., 2020). As a predominant subtype of cervical cancer, squamous cell carcinoma (SqCa) (Sahasrabuddhe et al., 2012) comprises 70% of all cases and typically follows a progression from HPV infection to low-grade squamous intraepithelial lesion (LSIL), then to high-grade squamous intraepithelial lesion (HSIL), with a process that may span over a decade (Gravitt and Winer, 2017). Thus, there is an urgent need for enhanced comprehension of the precancerous status. In 2024, Hu et al. collected HSIL/SqCa tissues from HPV-positive patients undergoing surgical biopsies to create a biobank containing cervical precancerous pathogens and tumor organoids, which retained genomic and transcriptomic profiles, as well as the causative HPV genome. Through coculturing the organoid models with HPV antigenic peptide-stimulated peripheral blood immune cells (Hu et al., 2024), different immune responses were observed in the two organoid models. This study established an experimental platform and biobank for conducting *in vitro* mechanistic studies on HPV-associated cervical diseases, screening therapeutic vaccines, and developing personalized treatment options.

Small cell carcinoma of the cervix (scCC) is also a rare and highly aggressive cancer associated with HPV. In a previous study, the organoids from a patient with HPV18-positive scCC were generated. Through whole exome sequencing and RNA-seq, therapeutic targets specific to HPV-derived scCC were identified. Additionally, utilizing organoids and organoid-derived mouse xenograft models, drug sensitivity testing was conducted. The findings all suggest the potential of tumor organoids in uncovering targets for rare cancers (Kusakabe et al., 2023).

## 5 Conclusions and prospects

**Figure 3:**
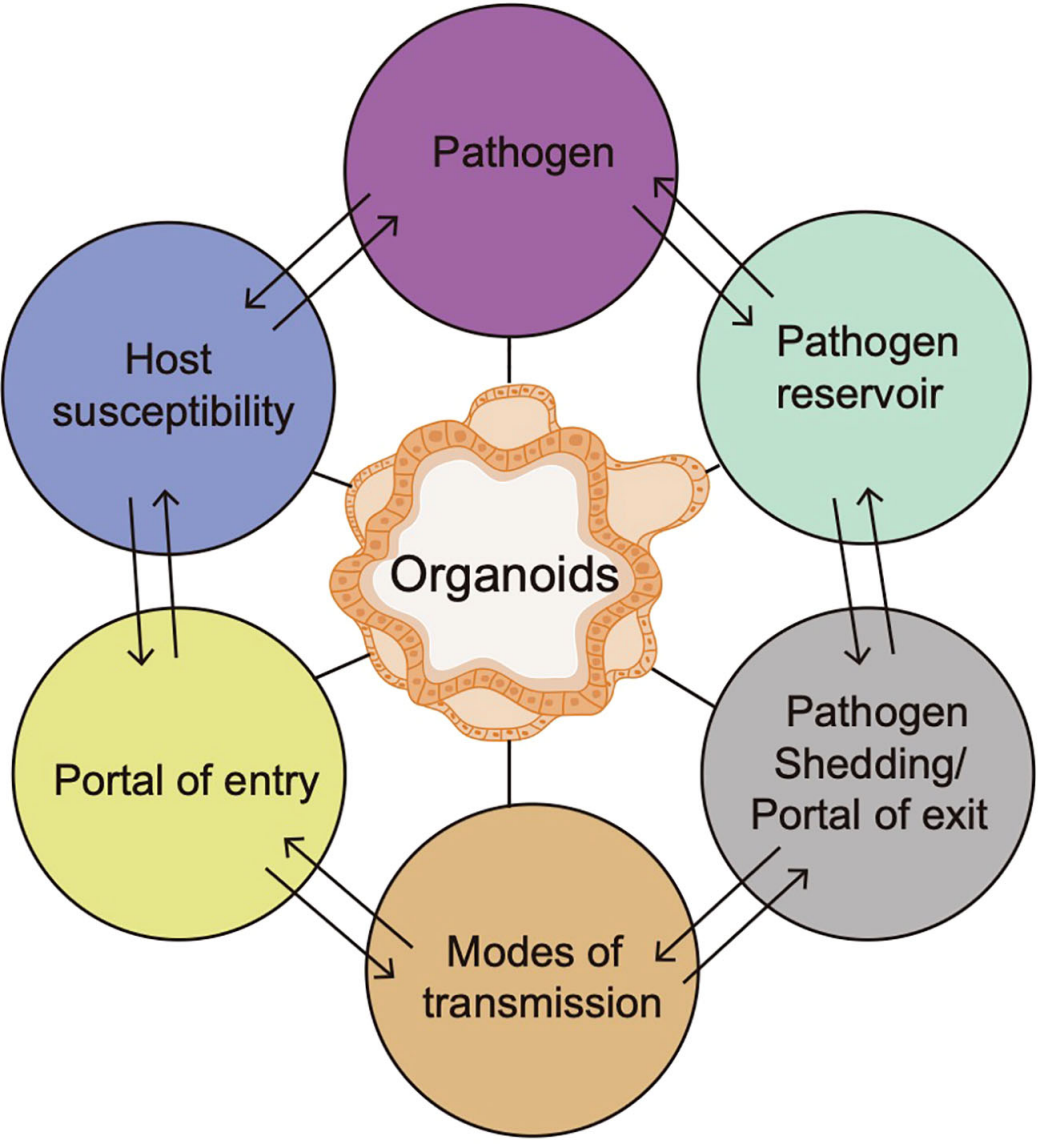
A schematic diagram of organoids to study diverse links of the chain of infection model.

Pathogenic infection may appear in various anatomical locations within the host, which is usually considered to be an inducement for diseases (Gilbert and Lewis, 2019). Due to infection, host-pathogen interactions can result in either host immunity or an aggravated immune response mainly based on 6 factors, including the host susceptibility, portal of entry, modes of transmission, portal of exit, pathogen reservoir and pathogens (Dutta and Clevers, 2017). Organoids, a platform for studying pathogen-induced tumorigenesis, can be used to study diverse links of the chain of infection model and help to develop more efficacious control measures against emerging pathogens, thus promoting the understanding of the host-pathogen interactions (**Figure 3**). Nevertheless, there are still several challenges that should be considered. First, the full impact of tumor microenvironment on tumor behaviors is difficult to be captured in organoids due to lack of stromal components. Second, the microbial colonization efficiency is variable, and the study of anaerobic bacteria requires specialized techniques, including specific culture methods and manipulation of microbes (Strobel, 2009). Moreover, high-throughput experimental setups are limited by the manual nature of the microinjection procedure (Bartfeld et al., 2015). Although the technique of directly coculturing pathogenic microorganisms with organoids at a specific multiplicity of infection (MOI) has been extensively employed, there remains a lack of standard protocols for MOI and infection timing. Notably, the mutations in organoids are typically subclonal, random, and primarily impact non-coding regions, but refinement and standardization of reagents and protocols for organoid culture are very necessary for their effective utilization in clinical settings, including precancerous study and beyond.

Notwithstanding these challenges, the coculture system of tumor organoids with pathogenic microorganisms is significant in comprehending and simulating the status of human viral infection, *in vivo* homeostasis, and disease progression. Outside the gastrointestinal tract, the microbiota can affect the immune function by regulating the balance of Treg cells, _γδ_T cells, and cytokine production. The brain interacts with the gastrointestinal system through a vast network described as the gut-brain axis, which may be expanded to include the gut microbiota, thus labeling the gut-microbiota-brain axis (Patterson et al., 2019). The existing preclinical data show that head injury can cause structural and functional damage to the digestive tract, but there is no experimental model that directly reflects this research (Sundman et al., 2017). Despite this gap, the coculture method proposed in this study may be used as a reference.

In the future, efforts will be made to gradually overcome the constraints above. The utilization of tumor organoid-based coculture models holds promise for enhancing patient-derived disease models, drug screening and stem cell research, as well as elucidating the interactions between pathogen-induced infection and tumor mechanisms, which paves the way for translational research and personalized treatment.
